# Uptake of Breathable Nano- and Micro-Sized Polystyrene Particles: Comparison of Virgin and Oxidised nPS/mPS in Human Alveolar Cells

**DOI:** 10.3390/toxics11080686

**Published:** 2023-08-10

**Authors:** Antonio Laganà, Giuseppa Visalli, Alessio Facciolà, Consuelo Celesti, Daniela Iannazzo, Angela Di Pietro

**Affiliations:** 1 Department of Biomedical and Dental Sciences and Morphofunctional Imaging, University of Messina, 98125 Messina, Italy; antonio.lagana1@studenti.unime.it (A.L.); giuseppa.visalli@unime.it (G.V.); alessio.facciola@unime.it (A.F.); 2 Istituto Clinico Polispecialistico C.O.T., Cure Ortopediche Traumatologiche s.p.a., 98124 Messina, Italy; 3 Department of Electronic Engineering, Industrial Chemistry and Engineering, University of Messina, 98125 Messina, Italy; consuelo.celesti@unime.it (C.C.); daniela.iannazzo@unime.it (D.I.)

**Keywords:** environmental wear, uptake, cytotoxicity, ROS overproduction, mitochondrial dysfunction

## Abstract

Airborne micro- and nanoplastics are widely spread and pose a risk to human health. The third polymer plastic most commonly produced and present in atmospheric fallout is polystyrene (PS). For these reasons and for a more realistic assessment of biological effects, we examined in-home oxidised (ox-, simulating photoaging) nPS/mPS (0.1 and 1 μm), comparing the effects with virgin ones (v-). On human alveolar cells (A549), we quantified the cellular uptake, using FITC-functionalised nPS/mPS, while cytotoxicity, changes in the acidic compartment, ROS production, mitochondrial function, and DNA damage were assessed to study the effects of internalised v- and ox-nPS/mPS. The results showed that the uptake was dose-dependent and very fast (1 h), since, at the lowest dose (1.25 µg/well), it was 20.8% and 21.8% of nPS and mPS, respectively. Compared to v-, significant ROS increases, DNA damage, and mitochondrial impairment were observed after exposure to ox-nPS/mPS. The enhancement of effects due to environmental aging processes highlighted the true potential impact on human health of these airborne pollutants.

## 1. Introduction

Plastics, due to their malleability, high versatility and low cost, are currently widely used, and their production is only slightly lower than that of concrete and steel [1]. Despite the remarkable durability, around 50% of the total mass of currently manufactured plastics is disposable. This aspect has greatly increased their presence in different environmental matrices, where improperly disposed plastic waste undergoes a slow and partial abiotic and biotic degradation. Mechanical action of wind and wave motions and prolonged exposure to ultraviolet (UV) light (photo-oxidation), as well as degradative microbiological processes), cause the fragmentation of plastic waste, producing secondary microplastics (<5 mm) and, following further fragmentation, secondary nanoplastics with a diameter ≤0.1 µm [2]. Primary microplastics and nanoplastics, intentionally and directly produced on a micro- and nanoscale as constituents of specific products, also contribute to plastic pollution [3]. Micro- and nanoplastics are persistent in the environment and can interact very easily with biological systems [4,5]. Indeed, they have been found in sediment [6], soil [7], seawater [8], high mountain lake ecosystems [9] and air [10], but also in many foods and beverages like shellfish, cooking salt, drinking water and beer [11]. Due to their hydrophobicity, micro- and nanoplastics are highly bioavailable and are able to bioaccumulate along the trophic scale [12,13]. 

Humans are inevitably exposed to micro- and nanoplastics, mainly via ingestion and inhalation [14,15,16,17,18,19], as confirmed by the presence of microplastics in different body regions as well as in faecal excretion [20,21,22,23,24].

Airborne micro- and nanoplastics are derived from a variety of sources, including synthetic fibres, waste disposal products, incinerators, agricultural practices (such as PS peat and sewage sludge used as fertilizer), as well as road traffic [10,25,26,27]. In particular, tyre wear particles (TWPs) and brake wear particles (BWPs) are formed by complex mixtures of metal and mainly microplastics. It has been estimated that, in ambient air, around 4% and 11% of the respirable (fine) and inhalable (coarse) particulate matter (PM) are, respectively, formed by micro- and nanoplastics derived from only TWPs and BWPs [28].

Moreover, indoor exposure to airborne micro- and nanoplastics appears to be highly relevant, considering the lower dilution volumes, the time spent in indoor environments (on average, 70–90% of our lifetimes) and the several sources of these pollutants (synthetic textile fibres, upholstery and furnishing objects or building materials) [29]. 

We currently know a lot about the pathogenesis of airborne micro- and nano-PM (fine and ultrafine PM), as well as metal- and carbon-based engineered nanoparticles [30,31,32]. The ≤2.5 µm particles can overcome mucociliary clearance and reach the alveolar surface, wherein nanoparticles can bypass the phagocytic activity of macrophages (alveolar clearance). They easily cross the pulmonary epithelial barrier and enter the bloodstream, from where they are distributed to various anatomical regions [33]. For micro- and nanoparticles, the number of surface atoms per unit mass is increased by several orders of magnitude, greatly enhancing the surface area for chemical reactions, while charge, polarity and the presence of surface reactive groups are fundamental in regulating cellular uptake and biological effects [34]. The pathogenesis of respirable micro- and nanoplastics is poorly known, but it can be assumed that the trigger is determined by oxidative stress, which causes lipid peroxidation, protein and DNA damage, mitochondrial dysfunction and inflammation in response to tissue damage [35].

Since the largest share of airborne micro- and nanoplastics that humans inevitably inhale are the secondary ones, subjected in the environment to a variable photo-oxidation, the aim of this in vitro study was to gain a more realistic assessment of the hazard by studying the biological effects of aged micro- and nanoplastics. In human alveolar epithelial cells (A549 cell line), the effects of in-home oxidised polystyrene nanoplastics and microplastics (ox-nPS/mPS), with diameters of 0.1 and 1 μm, were compared to virgin ones (v- nPS/mPS). In particular, after quantifying the uptake, the cytotoxic effects (i.e., viability loss, changes in acidic compartment, ROS production, mitochondrial impairment and DNA damage) in v- and ox-nPS/mPS were compared. The presence on the particle surface of carboxyl, alkoxyl and hydroxyl groups, simulating the photoaging due to ultraviolet (UV) radiation, induced by an in-home oxidative process, enhanced the particle reactivity, increasing the risk for the exposed subjects.

## 2. Materials and Methods

### 2.1. Exposure Conditions and Cell Models

Virgin nPS (average size 100 nm) and mPS (average size 1 µm) were purchased from Sigma-Merck (Milan, Italy; code: BCC8557 and BCC9279). The choice of diameters was based on the evidence that both mPS and nPS are part of the respirable fraction of inhalable particulate matter (≤2.5 μm).

nPS/mPS oxidation was performed by the method reported by Mielczarski et al. (2011) [36]. Briefly, to allow the presence of carboxyl, alkoxyl and hydroxyl groups on the particle surface, aliquots of stock suspensions in phosphate-buffered saline (PBS, 1:10 ratio) were treated at 80 °C for 2 h. The suspensions were characterised by Fourier-Transform Infrared (FT-IR) spectrometry, dynamic light-scattering (DLS), scanning electron microscopy (SEM) and UV-Vis spectrophotometry. As previously reported [37], dynamic light-scattering DLS analyses and SEM observations confirmed the same average size of the functionalised microplastics, suggesting that oxidation occurred only at the surface of the particles and that it did not cause their aggregation, either in PBS or in cell medium suspensions. UV-Vis and FTIR spectra highlighted the presence of oxygen functionalities, such as carbonyl and phenol groups. To quantify spectrofluorometrically the cellular uptake, these functionalised particles were covalently bound to fluorescein isothiocyanate (FITC). Specifically, the conjugation was performed using 1-ethyl-3-(3-dimethylaminopropyl) carbodiimide (EDC) and hydroxybenzotriazole (HOBt) as coupling reagents, and a polyethylene glycol (PEG) linker. This had two amino groups, one of which was previously bound to FITC, while the other was bound to functionalised particles (i.e., ox-mPS- and ox-nPS). The reaction steps, as well as the purification steps, were reported in detail in a previous study [37]. The complexes were then analysed by FTIR spectroscopy while the photoluminescence (PL) properties were investigated by dynamic light scattering (DLS) [37]. To assess the stability of the conjugates under the experimental conditions, a time course (1, 3, 6 and 24 h) was performed using mPS-FITC suspended in cell medium with 2% FBS and in saline solution at pH 6.5 (early endosomes) and 4.5 (mature endosomes). Briefly, after 1, 3, 6 and 24 h, the suspensions were centrifuged (12,000× *g* for 10 min) and a fluorometric reading was carried out (ex 485 nm, em 535 nm) in both supernatants and mPS resuspended in the same volume of fresh solutions. While the emission values were almost constant in FITC-mPS suspensions, no emissions were recorded in the supernatants, with the exception of the ones at pH 4.5 at 24 h. Compared with the respective suspensions, the emission values of the latter were within 10%.

The cellular uptake and the biological effects of virgin (v-) and oxidised (ox-) nPS/mPS suspensions were assessed in the human alveolar cell line A549 (ATCC-CCL-185 Tm), which is the model of choice for in vitro studies of airborne pollutants. The cells were cultured in F-12K medium (Gibco™ 21127022) supplemented with 2 mM of L-glutamine, 10% of inactivated foetal bovine serum (FBS), and 1% penicillin/streptomycin/amphotericin, at 37 °C in a 5% CO_2_/95% air humidified atmosphere. For all experiments, the exposure treatment for times and doses established in the experimental protocol was performed in semiconfluent monolayers grown for 24–36 h and incubated with nPS/mPS suspensions that were set up in cell medium containing 2% FBS (maintenance cell medium). Although the presence of proteins could partially neutralise the effect of the particles due to the so-called “protein corona”, we believe that this protocol best simulates what actually happens. The corona effect hinders the intake due to the particles’ increased bulk and the loss of hydrophobicity, which is known to promote the interaction of particles with cell membranes [38]. Moreover, the protocol meant that exposure to xenobiotics occurred under physiological conditions and not in cells which, kept in suboptimal conditions, could lead to an overestimation of the effects. Cells treated with PBS without nPs/mPS were used as a negative control while cells treated with suitable compounds were used as positive controls.

### 2.2. Cellular Uptake of nPS/mPS

The stock suspensions of nPS-FITC/mPS-FITC (80 µg mL^−1^) were diluted in culture medium and added (100 µL/well), in the range of 1.25–20 µg/well, to A549 cells that had been grown for 24 h in 96-well microplates (final density 4 × 10^4^ cells/well). After 0.5, 1, 3 and 24 h, fluorometric readings were carried out at the excitation and emission wavelengths of 485 nm and 535 nm, respectively, by using a microplate reader (Tecan Italia, Milan, Italy). After recording the emission values in each well, the medium was removed, the monolayer was washed repeatedly with PBS, to remove uninternalised particles, and emission values were recorded to measure the percentage uptake (i.e., intracellular nPS-FITC/mPS-FITC). Moreover, A549 monolayers grown in chamber slides were examined with confocal laser scanning microscopy (CLSM) using the Leica TCS SP2 instrument (Leica Microsystems, Wetzlar, Germany), with Leica Confocal software (version 2.0) used to process the images, which were acquired in both fluorescence and phase contrast. A Leica DM IRB fluorescence microscope (Leica Microsystems) was used to select the optical fields.

### 2.3. Viability Assays

In A549 cells, v-and ox-nPS/mPS-induced cytotoxicity was evaluated by using the colorimetric MTT assay, based on the reduction of 3,(4,5-dimethiazol-2)-2,5-difeniltetrazolium bromide, catalysed by cellular NAD(P)H-dependent dehydrogenases. Briefly, after verifying the absence of particle interference in the spectrophotometric detection of cell viability, the assay was performed in cells cultured for 24 h in 96-well microplates, to which the appropriate volume of stock suspensions in PBS (10 mg mL^−1^) was added in the medium. The tested concentrations ranged from 12.5 to 200 μg mL^−1^. Dimethyl sulphoxide (DMSO, 10%) was used as a positive control, and eight different wells were treated for each dilution. After following our standardised protocol [37], the enzymatic activity was quantified by spectrophotometric measurement at 540 nm, using a microplate reader (Tecan Italia). The optical density (OD) values obtained for each sample were compared to the mean OD of the negative control, which was arbitrarily considered corresponding to 100% viability.

### 2.4. Assessment of the Cellular Acidic Compartment

Changes in the endocytic apparatus (late endosomes and lysosomes), due to the uptake of virgin and oxidised nPS/mPS, were examined by employing metachromatic fluorophore Acridine Orange (AO), which is captured by protons and collected in the acidic compartment. Here, the highly concentrated probe will emit red fluorescence, while it will release green fluorescence in the cytosol and nucleus, where AO scarcely accumulates. The loss of red fluorescence is indicative of acidic compartment damage [39]. The analyses were performed in semi-confluent A549 monolayers grown in chamber slides and treated for 3 and 24 h at 37 °C with nPS/mPS suspensions (100 µg mL^−1^). After removing the medium and washing repeatedly with PBS, AO solution (5 µg mL^−1^) was added and CLSM was used to assess the endocytic apparatus and other morphological changes which were nPS/mPS-induced. To quantify the acid compartment, the image-processing program Image J (imagej.nih.gov/ij/index.html, accessed on 15 September 2022) was used to calculate the cellular area which emitted red fluorescence. These values were expressed as %, referring to the total area of each cell, and at least 100 cells were analysed for each slide.

### 2.5. Evaluation of ROS Production

To test the pro-oxidant effect of v- and ox-nPS/mPS, ROS were measured by using the 2′,7′-dichlorofluorescein-diacetate (DCF-DA) probe (Merck Life Science S.r.l., Milan, Italy). After crossing cell membranes by passive diffusion, the reagent is hydrolysed rapidly by cellular esterases to 2′,7′-dichlorofluorescein (DCFH). This non-fluorescent compound is oxidised in the presence of ROS, forming the highly fluorescent molecule 2′,7′-dichlorofluorescein (DCF). Briefly, after repeated washing with PBS, sub-confluent A549 monolayers (80%) in 96-well microplates were loaded with the probe solution (1 μM) prepared in PBS containing 10 mM of D-glucose (pH 7.4) and were incubated at 37 °C for 30 min [40]. After washing in PBS to remove the non-internalised probe, cells were treated with v- and ox-nPS/mPS suspensions (in the range of 25–200 μg mL^−1^). The fluorometric readings were carried out in the intervals of 0.5–24 h by using a microplate reader (Tecan Italia) at the excitation and emission wavelengths of 485 and 535 nm, respectively. ROS production was calculated as the percentage change (Δ%) compared with control cells.

### 2.6. Mitochondrial Transmebrane Potential

To assess mitochondrial impairment induced by v- and ox-nPS/mPS, we measured transmembrane potential by the incorporation of the fluorescent probe rhodamine 123 (R123) (Invitrogen Molecular Probes, Eugene, OR, USA). The chemical properties of the cationic fluorochrome R123 allow mitochondrial membrane crossing and storage in the matrix only in functional mitochondria that possess a transmembrane potential (ΔΨm), which is indicative of an active proton gradient maintained during oxidative phosphorylation [41]. A549 monolayers, grown in 96-well plates, were treated with 100 μg mL^−1^ of v- and ox-nPS/mPS suspensions for 24 h. After incubation at 37 °C and washing in PBS to remove the non-internalised particles, cells were treated with the probe solution (10 μM final concentration) and incubated for 10 min at 37 °C. Fluorimetric readings were carried out using a microplate reader (Tecan Italia, Milan, Italy) set to 535 and 595 nm as the excitation and emission wavelengths, respectively. In comparison to the control cells, the percentage changes of emission values were calculated for each sample.

### 2.7. Assessment of DNA Damage by the Comet Assay

A549 cells treated for 24 h with v- and ox-mPS/mPS suspensions (100 μg mL^−1^), were assessed for DNA integrity by using the alkaline version of the comet assay [42]. Tests were performed in duplicate on about 2 × 10^4^ cells for each spot, and the electrophoresis was carried out for 30 min at 300 mA and 25 V (0.86 V cm^−1^). The slides, stained with ethidium bromide (20 μg mL^−1^), were imaged using a DMIRB fluorescence microscope (Leica Microsystems), equipped with a digital camera (Power Shot S50; Canon, Milan, Italy), at 400× total magnification. Samples were run in duplicate, and images of 100 cells per slide were acquired randomly and analysed by using the Comet Assay Software Project (CASP) software (http://ww25.casplab.com/?subid1=20230810-1122-084e-8a7d-57f935e283f5 (accessed on 15 September 2022)). %TDNA (i.e., %DNA in the tail) was considered the parameter of DNA damage.

### 2.8. Statistical Analyses

All data are presented as the mean ± standard error (SE) based on at least three independent experiments. Analyses were performed using the Statistica programme (version 10). Lilliefors and Shapiro–Wilk normality tests were used to assess data distribution patterns. The relationships between different parameters were assessed by using the Pearson correlation coefficient, while the *t* Test was used to assess the differences between samples. Significance was accepted at *p* < 0.05.

## 3. Results

### 3.1. Cellular Uptakes of nPS/mPS

To spectrofluorimetrically quantify the uptake of nPS-FITC/mPS-FITC in A549 cells, we generated a time course of emission values. After ascertaining the absence of free FITC in the particle suspensions in cell medium in the interval 3–24 h, preliminary abiotic tests showed that the emission values (expressed in arbitrary fluorescence units [AFU]) under the experimental conditions were 97.13 and 72.45 for 1 µg of mPS and nPS, respectively. On this basis, in the range of 1.25–20 µg/well, we calculated the internalised amount (µg) at different times. After 1 h, at the lowest dose to which the cells were exposed, 20.8% and 21.8% of the amount of nPS and mPS, respectively, was internalised.

**Figure 1:**
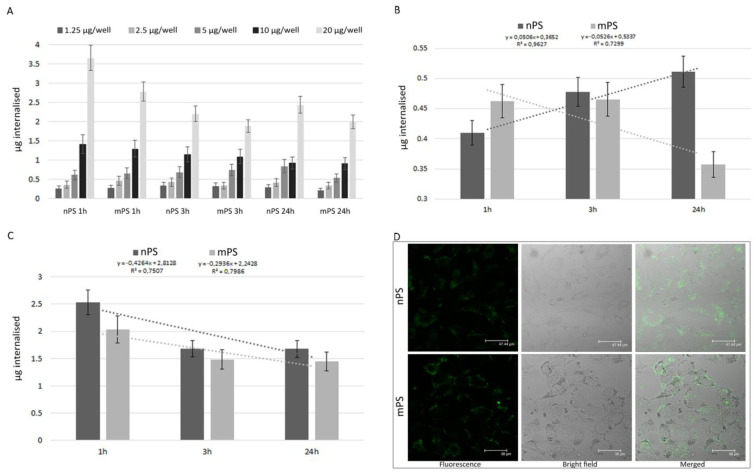
Cellular uptakes of PS/mPS. (**A**) Time course of spectrofluorimetric measurements of internalised µg of nPS-FITC/mPS-FITC in A549 cells treated in the range of 1.25–20 µg/well. Highly significant dose–effect correlation for both sizes of plastic particles was observed (*p* < 0.01 to Pearson test). (**B**,**C**) Trends of nPS-FITC/mPS-FITC uptake as a function of the exposure time in A549 cells exposed in the range of 1.25–2.5 µg/well (**B**) and in the range of 5–20 µg/well (**C**). In (**B**,**C**) each bar reports the average ± SE of internalised µg (based on FITC Emission value) recorded for 1.25 and 2.5 µg/well (lower doses) and 5, 10 and 20 µg/well of nPS-FITC/mPS-FITC, respectively. The graphs highlight the different trend over time of nPS-FITC/mPS-FITC uptake, showing a positive finding only for the lower concentrations of nPS. (**D**) CLSM images of nPS-FITC/mPS-FITC internalisation in A549 semiconfluent monolayers treated for 1 h at the dose corresponding to 10 µg/well. The cells exposed to nPS or mPS exhibited fair cytoplasmatic fluorescence, which was more intense in mPS-FITC-treated cells. In contrast microscopy image of these latter cells, intracytoplasmic aggregates of mPS were clearly visible.

The results in Figure 1A are expressed as internalised amounts (µg/well) of nPS/mPS and clearly highlight the significant dose–effect correlation for both sizes of plastic particles (Pearson correlation coefficient [r] > 0.99). On the other hand, nPS and mPS showed distinct trends as a function of the exposure time (Figure 1B,C). The amounts of internalised mPS decreased as the exposure time increased for all tested doses; the percentage decrease (Δ%) was between 20% (at the lower doses of 1.25–2.5 µg/well) and 30% (at the higher doses of 5–20 µg/well). For nPS, this trend was observed only at the higher exposure doses (Δ% > 30), while the uptake at lower doses increased by an average of 20% during the entire exposure period.

Considering the PS density and the size of the particles, we calculated the total number of internalised particles: ~200 mPS and 200,000 nPS per µg internalised. The markedly higher number of internalised nPS highlights the greater surface area developed by the last ones. Because the experiments were performed in 96-well microplates with an average of 4 × 10^4^ cells/well, regardless of the particle size, the uptake was in the range of 5.1–91.2 pg/cell.

CLSM confirmed the data and showed that the cells exposed to nPS/mPS exhibited fair cytoplasmatic fluorescence, which was clearly more intense in cells treated with the more fluorescent mPS (Figure 1D). In summary, the kinetics of nPS/mPS uptake indicated that internalisation was extremely fast, with a higher amount of internalised nPS.

### 3.2. nPS/mPS-Induced Changes of the Acidic Compartment

**Figure 2:**
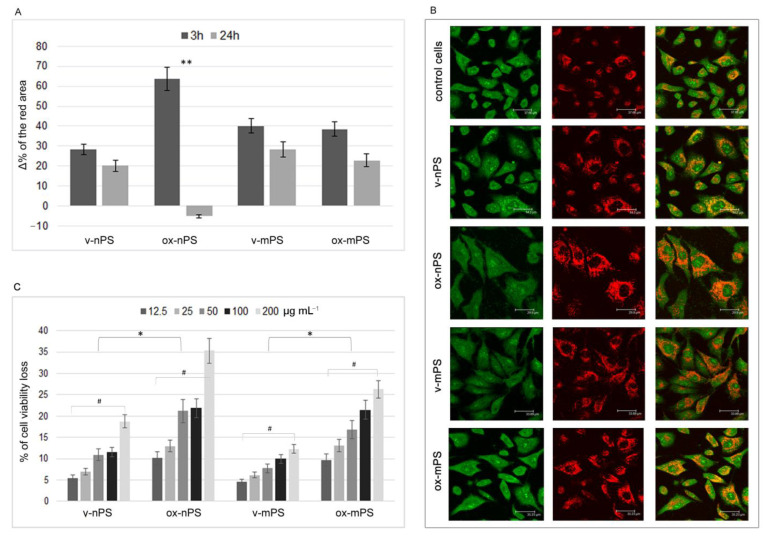
Changes in acidic compartment and cytotoxicity, v- and ox-nPS/mPS-induced. (**A**) The acidic compartment was assessed by employing metachromatic fluorophore AO which gives red colour when, due to the lower pH values, the dye builds up. The graph (**A**) reports Δ% of the area emitting red fluorescence in exposed cells in comparison to control cells. The experiments were performed in semi-confluent A549 monolayers grown in chamber slides and treated for 3 and 24 h at 37 °C with 100 µg mL^−1^ of v- and ox-nPS/mPS suspensions. A significant difference between 3 and 24 h was observed for ox-nPS (*p* < 0.01 to *t*-test). (**B**) Representative CLSM images in control and exposed cells (3 h). A549 cells exposed to v- and ox-nPS/mPS suspensions showed a very bulky acidic compartment made up of numerous red organelles that, particularly for ox-nPS, almost completely occupied the perinuclear cytosol. (**C**) Results of MTT assay in A549 treated for 24 h in the range of 12.5–200 µg mL^−1^, corresponding to 2.3 × 10^2^–3.68 × 10^3^ particles/well and 2.3 10^5^–3.68 × 10^6^ particles/well for mPS and nPS, respectively. In comparison to the virgin nPS/mPS, the percentages of cell viability loss were significantly higher in ox-nPS and mPS (* *p* < 0.05; ** *p < 0.01* to *t*-test). For all plastic particles, the decrease in cell viability was positively related to the exposure dose (# *p* < 0.01 to Pearson test). Compared to mPS, nPS showed a moderately higher cytotoxic effect.

The evaluation of the acid compartment gives an insight about the cellular ability to neutralize foreign particles. Therefore, employing the metachromatic fluorophore AO, the microscopic analyses of the endocytic apparatus in cells treated with v-nPS/mPS for 3 h highlighted a very bulky acidic compartment made up of numerous red organelles, clearly showing the consistent internalisation of both particles and, at the same time, the integrity of mature endosomes that almost completely occupied the perinuclear cytosol (Figure 2A,B).

Compared to control cells, in the v-nPS/mPS-treated cells the Δ% of the area which emitted red fluorescence was 28.3 and 40.1, respectively, on average. In contrast to mPS, for which the values of v- and ox- were superimposable, a significant increase in the phagosomal compartment was observed in cells treated with ox-nPS (Δ% 65.5). Further changes in the endocytic apparatus were observed after 24 h when, in the cells treated with both v-nPS and v- and ox-mPS, a moderate reduction in the area of the acidic compartment was observed. This, underlining a possible spill from the endocytic apparatus, was more evident in cells treated with ox-nPS in which acidic organelles were also less developed than the control cells (Δ% −5.1), and the differences compared to 3 h were highly significant (*p* < 0.01). The results obtained showed a greater alteration of the acid compartment in cells exposed to nPS than in mPS, which is more attributable to ox-nPS.

### 3.3. Cytotoxicity nPS/mPS-Induced

We evaluated cytotoxicity in our cell model by using the MTT assay. The tested doses ranged from 12.5 to 200 µg mL^−1^, corresponding to 2.3 × 10^2^–3.68 × 10^3^ particles/well and 2.3 × 10^5^–3.68 × 10^6^ particles/well for mPS and nPS, respectively, with an exposure time of 24 h. Both v-nPS and mPS had a moderate cytotoxic effect. Up to 200 µg mL^−1^, cell viability was >80% and, at the lowest exposure dose, cell viability was only about 5% lower than the control cells (Figure 2C). Unlike virgins, a more marked cytotoxicity was elicited by oxidised particles. In comparison to the virgin nPS/mPS, the percentage of cell viability loss was 1.9-fold and more than double in cells treated with ox-nPS and mPS, respectively (*p* < 0.05). For all plastic particles, the decrease in cell viability was positively related to the exposure dose. For v- and ox-nPS, the percentage of cell viability loss ranged between 5.4 and 18.8 and between 10.1 and 35.3, respectively (*p* < 0.01), while for mPS, these percentages ranged from 4.5 to 12.3 and from 9.7 to 26.3, respectively (*p* < 0.01). In conclusion, the assay underlined the moderately higher cytotoxic effect of nanosized particles, especially in the oxidised rather than in the virgin ones.

### 3.4. nPS/mPS Increased ROS Production

**Figure 3:**
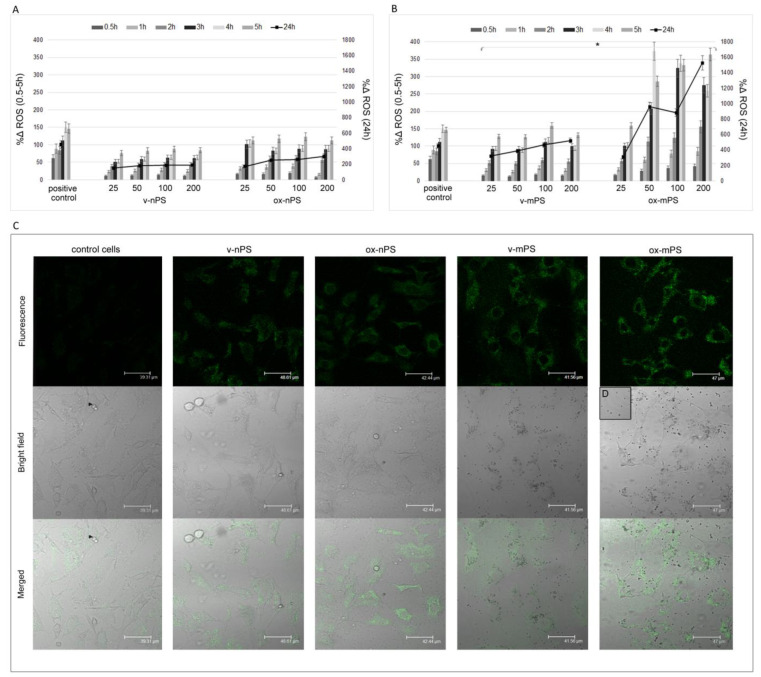
ROS overproduction induced by v- and ox-nPS/mPS. (**A**,**B**) Time courses (0.5–24 h) of ROS in A549 cells treated with nPS and mPS, respectively, in the range of 25–200 µg mL^−1^. H_2_O_2_ (300 µM) was used as positive control and the values are reported as %Δ relative to control cells. The two scales on the y axis show the values recorded in the interval 0.5–5 h on the left and those recorded at 24 h on the right. For v- and ox-nPS/mPS, exposure time and ROS values were always significantly related (r > 0.95; *p* < 0.01 to Pearson test). No dose effect was observed for v- nPS/mPS and for ox-nPS; conversely, ROS overproduction was positively related to doses (*p* < 0.01) for ox-mPS. Compared to v-nPS, significantly higher ROS values were observed for v-mPS (*p* < 0.05 to *t*-test). No significant differences were observed between v- and ox-nPS, while the pro-oxidant effect of ox-mPS was significantly higher compared to v-mPS (* *p* < 0.05). (**C**) CLSM images of the semiconfluent monolayers exposed to v- and ox-nPS/mPS (100 µg mL^−1^) for 3 h and treated with DCF-DA probe. A diffuse green cytosolic fluorescence, more intense in ox-mPS-treated cells, is shown. Moreover, as shown by (**D**) magnification of phase contrast micrograph, intracellular clusters of particles are clearly visible in the mPS-treated cells.

Figure 3A,B report the time course (0.5–24 h) of ROS production in A549 cells treated with v- and ox-nPS/mPS in the range of 25–200 µg mL^−1^. Similar to the kinetic uptake data, ROS overproduction was already evident after 0.5 h, especially for ox-nPS/mPS. Over time, ROS levels progressively increased, and r coefficients to the Pearson test were always >0.95 and similar to those calculated for the positive control (H_2_O_2_ 300 µM). In the interval of 0.5–24 h, DCF emission values increased on average by 15-fold for nPS and 17-fold for mPS, while no dose effect was observed for v-nPS/mPS. The pro-oxidant effect of v-mPS was significantly higher, with ROS values which were, on average, double compared to those of the v-nPS (*p* < 0.05) and only 20% lower than positive control. Compared to v-nPS/mPS, the increase was surprisingly smaller and equal to 40% for ox-nPS (P n.s). Conversely, ROS production induced by the ox-mPS was significantly increased, with emission values more than double (*p* < 0.05) in comparison to the v-mPS.

Moreover, ROS overproduction induced by ox-mPS was positively related to doses (*p* < 0.01), and the probe emission values were double compared to the positive control already at 50 µg mL^−1^ (Figure 3B).

CLSM observations of the semiconfluent monolayers of v- and ox-nPS/mPS-treated cells (100 µg mL^−1^) for 3 h confirmed the results (Figure 3C), highlighting diffuse green cytosolic fluorescence, which was more intense in mPS-treated cells. Moreover, as shown by magnification of phase contrast micrographs, intracellular clusters of particles are clearly visible in the mPS-treated cells (Figure 3D). In summary, ROS overproduction was very fast, size-dependent and higher in cells treated with oxidised particles.

### 3.5. nPS/mPS Induced Mitochondrial Dysfunction

**Figure 4:**
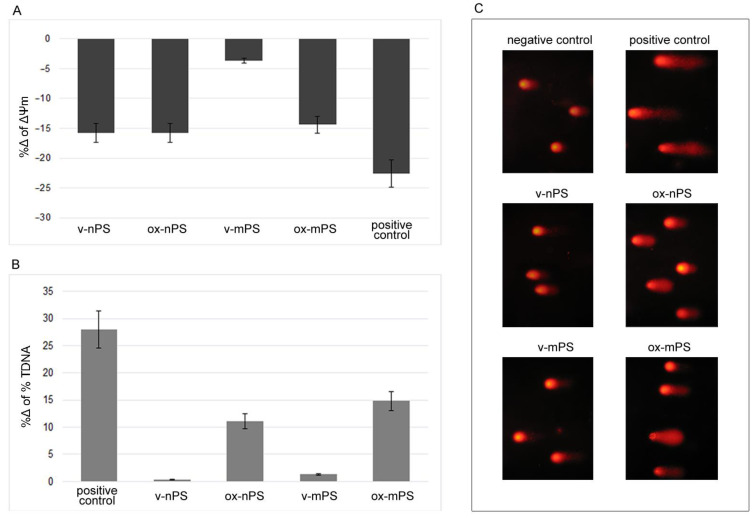
Mitochondrial impairment and DNA damage induced by v- and ox-nPS/mPS. (**A**) %Δ of transmembrane potential (ΔΨm) measured spectrophotometrically by the employment of R123. Conversely to v-mPS, both v- and ox-nPS and ox-mPS caused a moderate mitochondrial impairment (%Δ 16 vs. control cells). (**B**) Results expressed as %Δ of % TDNA to comet assay. The test was performed after overnight exposure to 100 µg mL^−1^ suspensions of v- and ox-nPS/mPS. H_2_O_2_ (300 µM) was used as positive control. Unlike v- nPS/mPS, the ones oxidised caused a moderate increase in DNA damage. (**C**) Representative images of comet assay that was replicated three times with similar results. Compared to v- nPS/mPS-treated cells, higher level of DNA damage is observed in ox-nPS/mPS-treated cells.

Transmembrane potential (ΔΨm) was detected to assess mitochondrial dysfunction induced by v- and ox-nPS/mPS. The experiments (Figure 4A) highlighted a moderate decrease in nPS-treated cells (%Δ 16 vs. control cells), without differences between v- and ox-nPS/mPS. Almost the same decrease was recorded in cells treated with ox-mPS. Instead, the virgin counterpart of these micro-sized particles did not alter mitochondrial function (%Δ −5 vs. control cells), clearly highlighting that ROS overproduction induced by v-mPS was directly attributable to the particles and not secondary to mitochondrial dysfunction. In summary, v-/ox-nPS and ox-mPS caused a modest mitochondrial dysfunction in treated cells.

### 3.6. nPS/mPS-Induced DNA Damage

The genotoxicity of v- and ox-nPS/mPS was assessed by the comet assay performed after overnight exposure to 100 µg mL^−1^ suspensions. H_2_O_2_-treated cells (300 µM) served as the positive control. In our cell model, v-nPS/mPS did not cause DNA damage and the %TDNA values almost completely overlapped with the control cells (Figure 4B,C). Conversely, both the oxidised particles were genotoxic and, compared to positive control, the %TDNA values were only 15% (nPS) and 10% (mPS) lower.

The results were consistent with ROS overproduction and highlight the oxidative DNA damage induced by exposure to oxidised nPS/mPS.

## 4. Discussion

The potential adverse health effects of breathable micro- and nanoplastics in humans are still poorly studied, despite the increasing quantities of airborne micro- and nanoplastics that can be found in the ambient air and, above all, in indoor environments. Several factors contribute to make plastic air pollution a threat for human health. These include both the features of micro- and nanoplastics and the anatomical and physiological characteristics of humans.

As reported in the introduction, the progressive fragmentation of plastics and their low density favour their long stay in the air (fly particles). Other intrinsic features of the plastic particles, such as hydrophobicity, favour a closer interaction with cell membranes, inducing higher translocation rates [10].

Regarding humans, we must consider how the defence mechanisms of the respiratory system are undoubtedly less efficient against microparticles (≤2.5 µm) and, above all, nanoparticles (≤ 0.1 µm). In particular, while microparticles are phagocytosed by alveolar macrophages, a process which is much slower than mucociliary clearance and able to trigger the inflammatory cascade, nanoparticles may bypass macrophage clearance. To maximise gas exchange, the alveolar epithelium is extremely extended (about 140 m^2^), and the alveolar–capillary barrier is particularly thin (<1 µm), which greatly favours the ability of nanoparticles to cross it and enter the bloodstream. Moreover, considering the volumes of air breathed daily (always >10 m^3^ in adults), even a reduced presence of airborne micro- and nanoplastics (a single particle L^−1^ corresponds to 10,000 respired particles) would cause their accumulation both in the lungs and, via the bloodstream, in other organs, triggering pathogenic processes.

Despite some limitations, mainly due to the lack of characterization of nPS/mPS in the biological environment where proteins and other components can modify the sizes and the cell-particle interactions, our in vitro study improves our knowledge of the effects of inhaled airborne micro- and nanoplastics. We highlighted the enhanced damage attributable to the surface changes of the particles due to oxidative processes, undergone during their environmental stay. As previously reported [37], the “artificial aging” used by us significantly increased the presence of carboxyl, alkoxyl and hydroxyl groups on their surfaces, simulating the photoaging endured by the particles once released into the environment [43]. The increase in oxygen-containing groups after the oxidative process was confirmed by Fourier-Transform Infrared (FT-IR) spectrometry, dynamic light-scattering (DLS), scanning electron microscopy (SEM) and UV-Vis spectrophotometry [37], which coincided with that which was reported by Biale et al. (2021) [44], showing surface-limited formation of oxidised aromatic structures in PS particles without any involvement of the overall polymer mass. As assumed, the presence of oxygen-containing groups on the particle surface increased the reactivity, and our results were confirmed by several studies [45,46,47,48].

By using homemade FITC-loaded nPS/mPS, we quantified the uptake and highlighted the speed at which both nPS and mPS were internalised. Similar to that which has been demonstrated for other particles [30], hydrophobicity favours the close interaction between micro- and nanoparticles and cell membranes, justifying both the speed of the process and the yield, as confirmed by the internalised doses, which ranged from 5.1 to 91.19 pg/cell. For both nPS and mPS, the process was significantly dose-dependent, while only the internalisation of nPS at low doses was time-dependent. The observed internalisation rate, equal to approximately one fifth at low exposure doses, and the volumes of air breathed daily (always >10 m^3^ in adults), clearly underscore the potential impact of these emergent pollutants on human health. Even a reduced presence of airborne micro- and nanoplastics (a single particle L^−1^ corresponds to 10,000 respired particles daily) would cause their accumulation both in lungs and, via the bloodstream, in other organs, triggering pathogenic processes. In humans, the defence mechanisms of the respiratory system are undoubtedly less efficient against microparticles (≤2.5 µm) and, above all, nanoparticles (≤0.1 µm). In particular, while microparticles are phagocytosed by alveolar macrophages, a process which is much slower than mucociliary clearance and able to trigger the inflammatory cascade, nanoparticles may bypass macrophage clearance. To maximise gas exchange, the alveolar epithelium is extremely extended (~140 m^2^ in adults), and the alveolar–capillary barrier is particularly thin (<1 µm), which greatly favours the ability of nanoparticles to cross it and enter the bloodstream.

Despite endocytosis being the main pathway of particle internalisation in all cells, we cannot exclude the possibility that nanoparticles can cross cell membranes via the energy-independent diffusion process. Diffusion is gradient-dependent and, albeit partially, is counteracted by the frictional coefficient of the particle that is in turn related to both the medium viscosity and the interactions between particles and macromolecules diluted in the solvent [49,50]. 

Considering energy-dependent endocytosis, our cell models allowed us to verify internalisation in epithelial alveolar cells (pinocytosis) which involves actin polymerisation, as shown by Varma et al. [51]. Therefore, the process requires GTPase activity and can be receptor-mediated (clathrin-dependent endocytosis or caveola-mediated endocytosis) [50]. The role of the endocytosis pathway had been confirmed in two models of intestinal cells by using inhibitors of caveola- and clathrin-mediated endocytosis [51,52]. After membrane invagination, the particles are internalised in early endosomes, which merge with lysosomes to form endolysosomes (late endosomes); our results revealed the fast load (i.e., 3 h) of v- and ox-nPS/mPS in the acidic compartment, highlighted by the enlargement of endolysosomes. Extending observation times, the reduction of the acid compartment, particularly evident for ox-nPS, could be attributed to endolysosomal permeabilisation, producing irreversible cytoplasmic acidification, enzymolysis and apoptosis. As confirmed by the lower viability recorded by the MTT test, the endolysosomal permeabilisation induced by ox-nPS was more marked, while the moderate cytotoxicity leads us to believe that a limited number of cells were involved in this effect after exposure to the v-nPS/mPS and to ox-mPS.

Despite the massive seizure in the endocytic apparatus, a share of nPS/mPS is randomly localised in the cell cytoplasm, causing the observed oxidative damage. We have reported similar results in HT-29 cells exposed to 3 and 10 µm PS particles [17], and similar results were obtained in Caco-2 cells [53].

For both nPS and mPS, the cellular-induced redox imbalance was time-dependent and higher in cells treated with oxidised mPS, confirming their intracellular bioavailability and the most powerful pro-oxidant effect of aged particles. Surprisingly, the redox imbalance of aged particles was more evident for mPS, despite the higher surface/mass ratio of the nPS, which notably increases reactivity [32]. Conceivably, this result is imputable to the higher cytotoxicity elicited by ox-nPS. The detachment of a large number of cells with internalised nPS did not allow us to assess oxidative damage in its entirety.

The most powerful pro-oxidant effect of aged particles was highlighted by the results of the comet assay and mitochondrial transmembrane potential and, unlike the virgin counterpart, oxidised nPS/mPS was able to cause DNA damage and mitochondrial dysfunction.

Since oxidation is the most important degradation process which plastics undergo during their aging in the environment, our results highlight in a more realistic way the potential health risk of the general population, mainly exposed to aged nPS/mPS, downsizing the value of the first in vitro studies almost always performed using virgin micro- and nano-polystyrene particles. However, it should be emphasised that nPS impaired mitochondrial function, regardless of whether it was virgin or oxidised. The decreases in transmembrane potential after exposure to v- and ox-nPS confirmed the results of Wu et al. (2019) [53] who observed transmembrane depolarisation in Caco-2 cells exposed to virgin nanoplastics. In addition to nPS, ox-mPS also decreased transmembrane potential, confirming that mitochondrial impairment, by triggering a vicious circle, further contributes to ROS overproduction, which was significantly increased in cells exposed to ox-mPS. Moreover, considering the key role played by mitochondria in triggering apoptosis [54], the observed mitochondrial impairment is certainly not to be underestimated in outlining the pathogenetic mechanism of these emergent airborne pollutants.

## 5. Conclusions

Overall, our results highlight the potential negative effects of the respirable fraction of plastic particles on human health. These airborne particles, remaining in the environment for relatively prolonged times, undergo numerous degradation processes, including photochemical ones that cause oxidation, increasing their reactivity. Simulating the photoaging process and comparing the effects induced by oxidised nPS/mPS to those of virgins, we clearly demonstrated that the greatest damage is elicited by the former, underlining the importance of performing the risk assessment using environmentally aged particles. Although the effects induced by these airborne pollutants are much less powerful than those of other airborne particles (combustion by-products, metals, engineered nanoparticles, etc.), their potential impact on human health cannot be excluded, especially if urgent action is not taken to limit their presence in the environment, significantly increased also due to massive use of face masks during the SARS-CoV-2 pandemic.

## Data Availability

Data is contained within the article.
